# Ultra-Short Antimicrobial Peptoids Show Propensity for Membrane Activity Against Multi-Drug Resistant *Mycobacterium tuberculosis*

**DOI:** 10.3389/fmicb.2020.00417

**Published:** 2020-03-17

**Authors:** Jasmeet Singh Khara, Biljana Mojsoska, Devika Mukherjee, Paul R. Langford, Brian D. Robertson, Håvard Jenssen, Pui Lai Rachel Ee, Sandra M. Newton

**Affiliations:** ^1^Department of Pharmacy, National University of Singapore, Singapore, Singapore; ^2^Section of Paediatric Infectious Disease, Department of Infectious Disease, Imperial College London, London, United Kingdom; ^3^Department of Science and Environment, Roskilde University, Roskilde, Denmark; ^4^MRC Centre for Molecular Bacteriology and Infection, Department of Infectious Disease, Imperial College London, London, United Kingdom

**Keywords:** tuberculosis, peptoids, drug resistant, membrane, anti-mycobacterial, *Mycobacterium tuberculosis*

## Abstract

Tuberculosis (TB) results in both morbidity and mortality on a global scale. With drug resistance on the increase, there is an urgent need to develop novel anti-mycobacterials. Thus, we assessed the anti-mycobacterial potency of three novel synthetic peptoids against drug-susceptible and multi-drug resistant (MDR) *Mycobacterium tuberculosis in vitro* using Minimum Inhibitory Concentration, killing efficacy and intracellular growth inhibition assays, and *in vivo* against mycobacteria infected BALB/c mice. In addition, we verified cell selectivity using mammalian cells to assess peptoid toxicity. The mechanism of action was determined using flow cytometric analysis, and microfluidic live-cell imaging with time-lapse microscopy and uptake of propidium iodide. Peptoid BM 2 demonstrated anti-mycobacterial activity against both drug sensitive and MDR *M. tuberculosis* together with an acceptable toxicity profile that showed selectivity between bacterial and mammalian membranes. The peptoid was able to efficiently kill mycobacteria both *in vitro* and intracellularly in murine RAW 264.7 macrophages, and significantly reduced bacterial load in the lungs of infected mice. Flow cytometric and time lapse fluorescence microscopy indicate mycobacterial membrane damage as the likely mechanism of action. These data demonstrate that peptoids are a novel class of antimicrobial which warrant further investigation and development as therapeutics against TB.

## Introduction

Tuberculosis (TB), caused by *Mycobacterium tuberculosis*, is the leading cause of death among infectious diseases according to the World Health Organization (WHO). In 2017, 10 million people were diagnosed with TB, while 1.6 million cases result in death (WHO, 2018). In addition, there were 446,000 cases of multi-drug resistant TB (MDR TB) and 558,000 cases were resistant to rifampicin alone, the standard first-line anti-mycobacterial. The shortfalls associated with the current TB therapy are the long duration of treatment leading to non-adherence to the regimen and the emergence of drug resistant strains, and failure to completely cure cases of MDR or extensively drug resistant (XDR) TB (Mukherjee et al., 2016). This problem is compounded by the rise in drug resistance and the concomitant decrease in available drugs. With only Bedaquiline and Delamanid approved in the last few years (Esposito et al., 2015), there is an urgent and unmet need for the development of novel anti-mycobacterial agents against drug resistant TB to stop the global epidemic.

Antimicrobial peptides (AMPs) are short, amphipathic, cationic molecules. They have been widely studied as most bacteria, including mycobacteria, have an extremely low propensity to develop resistance against them due to their non-specific mode of action (Gordon et al., 2005; Zhang and Gallo, 2016; Katarzyna and Małgorzata, 2017; Kang et al., 2017). Their main mode of action is to act by lysing or disrupting the bacterial membrane, although some AMPs are able to pass through the lipid bilayer without permeabilization and exhibit intracellular effects such as inhibition of cell wall, nucleic acid and protein synthesis (Haney et al., 2017). Their broad spectrum of activity against a wide range of microorganisms and rapid mode of action combined with the low proclivity for resistance development makes them an attractive choice for therapeutics against drug resistant organisms (Ghosh et al., 2014). However AMPs have various drawbacks, including their sensitivity to enzymatic degradation (Miller et al., 1994), low bioavailability and the high costs of synthesis (Gordon et al., 2005; Khara et al., 2016). These shortcomings make the translation of AMPs as therapeutics for drug resistant infections from bench to bedside a challenging undertaking.

To overcome the disadvantages of AMPs, oligo-N-substituted glycines (peptoids) are a good alternative (Czyzewski et al., 2016). Peptoids are sequence-specific peptidomimetics (Simon et al., 1992) with a peptide backbone but differ from AMPs in that the side chains are attached to the amide nitrogen instead of the α-carbon (Godballe et al., 2011); thus, there are no known proteases that will recognize and degrade the peptoid structure making them more stable. Furthermore, any chemical group which can be used as a primary amine can be incorporated in to a peptoid via submonomer synthesis thus giving rise to a much larger library of compounds with greater variation in side chains for development than is possible by modifying conventional AMPs. Interest in peptoids has increased over the past few years due to their antimicrobial activity against a broad spectrum of pathogens, non-specific mode of action, decreased susceptibility to enzymatic degradation, stability to heat and the relative ease of synthesis (Godballe et al., 2011; Mojsoska et al., 2017). Peptoids have been shown to act via disruption of bacterial membranes and have increased membrane permeability when compared to peptides, or by interacting with intracellular targets such as bacterial DNA (Miller et al., 1994; Mojsoska et al., 2017). Thus, peptoids have great potential to be developed into novel therapeutic adjuncts to existing drug regimens for TB.

Structure-activity relationship studies (SAR) reveal that tuning the structure of peptoids is an important key process when designing potent antimicrobials. For example, Mojsoska et al. (2015) designed a library of short linear cationic and hydrophobic peptoids with modifications to study the effect of hydrophobicity on peptoid activity and cytotoxicity whilst maintaining a constant charge. They found that higher hydrophobicity resulted in greater potency against *Staphylococcus aureus* but not against *Escherichia coli* or *Pseudomonas aeruginosa in vitro*. They also showed that the introduction of aromatic residues resulted in the loss of selectivity between bacterial and mammalian membranes. Other studies on antimicrobial peptoids against Gram positive and Gram negative bacteria have shown promising results (Bremner et al., 2010; Ghosh et al., 2014; Bolt et al., 2017). With regards to the anti-tubercular activity of peptoids, there has been only one study by Kapoor et al. that demonstrated potency against *M. tuberculosis* exerted by short lipophilic (Ntridec) peptoids compared to peptoids with shorter or no lipophilic tail attached. They evaluated the efficacy and cytotoxicity of their peptoids but did not shed any light on the mechanism of action of these compounds (Kapoor et al., 2011).

In this study we used a whole cell screening approach to determine whether peptoids can be a good alternative to AMPs to tackle the global epidemic caused by TB. Peptoids were designed with repeating monomeric units mimicking three amino acids (*N*lys, *N*spe, *N*he) to study the effect of chain length on peptoid activity against *M. tuberculosis*. Peptoids mimic the best features of AMPs, i.e., hydrophobicity, cationic nature and amphipathicity while overcoming their disadvantage of being susceptible to protease degradation (Bolt et al., 2017). Thus, by incorporating these characteristics into our design we could begin to determine the influence of positively charged, hydrophobic and aromatic chiral residues on the biological activity of the peptoids. We evaluated the *in vitro* anti-mycobacterial activity of the peptoids against both drug sensitive and MDR mycobacteria using the broth microdilution method. We examined the cytotoxicity against the murine macrophage cell line, RAW267.4, and evaluated the selectivity index (SI) for the peptoids. Furthermore, we assessed the killing efficacy in broth and mycobacteria infected macrophages as *M. tuberculosis* is primarily an intracellular pathogen. As some AMPs can modulate the host immune response (Dawson and Scott, 2012), we also evaluated the ability of the peptoids to activate macrophages. The mechanism of action of peptoids against MDR *M. tuberculosis* was studied using flow cytometric analysis and time lapse microscopy with propidium iodide (PI) uptake. Finally, we evaluated the *in vivo* efficacy of our lead peptoid, BM 2, *in vivo* using BALB/c mice.

## Materials and Methods

### Ethics

All animal procedures were performed under the license issued by the UK Home Office (PPL/708653) and in accordance with the Animal Scientific Procedures Act of 1986. BALB/c mice (Charles River Ltd, United Kingdom) were maintained in biosafety Containment Level (CL)-3 facilities according to institutional protocols.

### Media and Reagents

Dulbecco’s Modified Eagle Medium (DMEM), Phosphate Buffered Saline (PBS), rifampicin, moxifloxacin, dimethyl sulfoxide (DMSO), Tween 80, glycerol, PI, HPLC-grade water, Triton X-100 and lipopolysaccharide (LPS) from *Escherichia coli* 0111:B4 were obtained from Sigma-Aldrich (St Louis, MO, United States). Fetal Bovine Serum (FBS) was obtained from Labtech International (Sussex, United Kingdom). Middlebrook 7H9 broth, Middlebrook 7H11 agar, BBL Middlebrook Albumin Dextrose Catalase (ADC) supplement and Oleic Acid, Albumin, Dextrose, Catalase (OADC) were purchased from BD (Sparks, MD, United States). MTT [3-(4,5-dimethylthiazol-2-yl)-2,5-diphenyltetrazolium bromide] was from Duchefa Biochemie (Haarlem, Netherlands). The Griess Reagent System was from Promega (Madison, WI, United States).

### Peptoid Synthesis

The peptoids were synthesized by submonomeric solid phase synthesis as described by Mojsoska et al. (2015). Briefly, peptoid sequences were synthesized using an automated (Intavis, ResPep SL Bioanalytical Instruments AG) synthesizer on Rink amide MBHA resin on a 15 μM scale. Peptoids were cleaved from the resin in a trifluoroacetic acid (TFA)-water-triisopropylsilane (95:2.5:2.5) solution for 30–60 min and purified (>95%) using reverse-phase HPLC on a C_18_ column (10 μm, 250 × 10 mm; Higgins Analytical, Inc.) with an acetonitrile-water gradient (0.1% TFA). Upon purification, no TFA adducts were observed. The analytical purity and correct mass were verified using an analytical Dionex UltiMate 3000 reverse-phase UPLC (Thermo Scientific^TM^) with a C_18_ (100 Å, 100 × 2.1 mm; Kinetex) and electronspray ionization mass spectrometry (Finnigan LTQ). The mass spectra of the peptoids are shown in Supplementary Figure S1 and Supplementary Table S1.

### Mycobacterial Strains and Growth Conditions

*M. tuberculosis* H37Rv was obtained from the ATCC (United States). MDR clinical isolate, *M. tuberculosis* CSU87, resistant to rifampicin, isoniazid, ethambutol, streptomycin and kanamycin, and *Mycobacterium bovis* BCG *lux* (BCG) were gifts from Dr. Diane Ordway, Colorado State University and Professor Douglas Young, Imperial College London, respectively. Liquid cultures were grown in Middlebrook 7H9 broth, supplemented with 0.05% Tween 80, 0.2% glycerol and 10% ADC, to mid-log phase at 37°C in a shaking incubator (180 rpm) (Khara et al., 2016). Mycobacterial colonies were grown on Middlebrook 7H11 agar supplemented with 0.5% glycerol and 10% OADC. BCG were grown in broth or on agar, supplemented with hygromycin (50 mg/L) as a selection marker.

### Growth Inhibition and Minimum Inhibitory Concentration (MIC) Assays

The anti-mycobacterial activities of the peptoids were assessed using the standard broth microdilution method. Mid-log-phase bacterial cultures were diluted to 10^6^ colony forming units (CFU)/mL and 100 μL was added to twofold serial dilutions of the compounds in a 96-well plate. Following incubation at 37°C in a shaking incubator for 7 days, optical density (OD_595_) was measured. The lowest concentration with no bacterial growth was defined as the MIC, determined visually and by spectrophotometric measurements using the iMark^TM^ Microplate Absorbance Reader (Bio-Rad Laboratories, Hertfordshire, United Kingdom) (Khara et al., 2014, 2016).

### *In vitro* Killing Efficacy

Peptoid BM 2 was evaluated for its bactericidal activity against H37Rv and CSU87 by serially diluting with broth to give concentrations of 4 (0.5 × MIC), 8 (1 × MIC), 16 (2 × MIC) and 32 mg/L (4 × MIC) for H37Rv and 2 (0.5 × MIC), 4 (1 × MIC), 8 (2 × MIC), 16 mg/L (4 × MIC) for CSU87. Bacterial culture (100 μL), corresponding to an inoculum size of 10^6^ CFU/mL, was added to an equal volume of peptoid and incubated shaking for 7 days at 37°C. Samples were plated on agar and CFU were enumerated (Khara et al., 2016).

### Cell Culture and Cytotoxicity

Peptoids were assessed for cytotoxic effects on the mouse macrophage cell line (RAW 264.7) by the 3-(4,5-dimethylthiazol-2-yl)-2,5-diphenytetrazolium bromide (MTT) cell viability assay. Cells were maintained in DMEM supplemented with 10% FBS and cultured at 37°C in 5% CO_2_. Cells were seeded in 96-well plates (1 × 10^4^ cells per well) and incubated for 24 h before treating with concentrations of peptoids, up to 64 mg/L, for 24 h. Next, the medium was replaced with DMEM and MTT solution (5 mg/L) and incubated at 37°C for 4 h. Formazan crystals were dissolved in DMSO and OD was measured spectrophotometrically at 595 nm in a VersaMax Tunable microplate reader (Molecular Devices, Sunnyvale, CA, United States). Cell viability was determined relative to untreated controls and expressed as (*A*_595_ of treated sample)/(*A*_595_ of control) × 100% (Khara et al., 2016).

To determine peptoid selectivity for activity against bacterial over mammalian cells, the selectivity index (SI) was calculated as the ratio between the IC_50_ (concentration that inhibits 50% of metabolic activity of RAW 264.7 cells) and MIC.

### Nitric Oxide (NO) Production by Peptoid-Treated Macrophages

To evaluate the stimulation of macrophages, RAW 264.7 cells were seeded at 4 × 10^4^ per well in microtiter plates and incubated at 37°C in 5% CO_2_ for 24 h. Cells were then treated with fresh medium containing peptoids (doubling concentrations ranging 2–64 mg/L) for 24 h. The production of NO was estimated using nitrite as a surrogate and measuring nitrite concentrations in supernatants using the Griess reagent (0.1% *N*-1-napthylethylenediamine dihydrochloride, 1% sulfanilamide and 5% phosphoric acid). The absorbance was measured at 540 nm and nitrite concentrations were determined using standard curves generated with NaNO_2_ solutions. Macrophages stimulated with LPS (0.0001 mg/L) served as positive controls, while unstimulated macrophages served as negative controls (Khara et al., 2016).

### Intracellular Anti-mycobacterial Activity

The intracellular activity of peptoid BM 2 was assessed against H37Rv and CSU87 as described previously (Wilkinson et al., 1999). Briefly, RAW 264.7 cells were plated in to 96-well microtiter plates at a final concentration of 4 × 10^4^ cells per well and incubated for 24 h. Bacterial cultures were washed, resuspended in DMEM and added at a final concentration of 4 × 10^5^ CFU per well to achieve a multiplicity of infection (MOI) of 10:1. Plates were incubated at 37°C and 5% CO_2_ for 4 h and cells were washed with pre-warmed DMEM thrice to remove extracellular bacteria. BM 2, suspended in DMEM, was tested in triplicate at 4, 8, 16, 32 mg/L corresponding to 0.5 ×, 1 ×, 2 ×, 4 × MIC for H37Rv and 1 ×, 2 ×, 4 ×, 8 × MIC for CSU87. Macrophages were then lysed with sterile water, and the samples plated on agar at time 0 and 4 days for CFU determination (Wilkinson et al., 1999; Khara et al., 2016).

### Flow Cytometric Analysis of Membrane Permeabilization

To determine whether BM 2 was membrane active, the integrity of the mycobacterial membrane was evaluated using PI treated cells via flow cytometry. Briefly, H37Rv and CSU87 were washed, resuspended to give a cell density of 10^8^ CFU/mL, and then incubated in the presence of the peptoids (4 × MIC) for 3 h. Next, cells were treated with 20 mg/L of the membrane impermeable dye, PI, by incubating the cultures for 30 min at 4°C, followed by washing to remove unbound dye. Flow cytometric analysis was performed using a CyAnTM ADP Analyzer (Becton Dickinson, San Jose, CA, United States). Rifampicin and moxifloxacin (4 × MIC), served as negative controls.

### Microfluidic Live-Cell Imaging With Time-Lapse Fluorescence Microscopy

Live-cell imaging was performed using the automated CellASIC ONIX Microfluidic Platform with CellASIC ONIX B04A-03 Microfluidic Bacteria Plates (EMD Millipore Corporation, Hayward, CA, United States) using BCG due to the housing of the platform being in a CL-2 laboratory. BCG cultures were diluted with broth to 10^7^ CFU/mL and 100 μL was added to each cell inlet wells. BM 2 was serially diluted with broth (4 × MIC) and added with PI (10 mg/L) to the inlet wells (total volume 350 μL). The microfluidic plate was vacuum-sealed to the F84 manifold and the CellASIC ONIX FG Software initiated. Loading and washing of un-trapped bacterial cells was carried out according to the manufacturer’s protocol. Peptoid was perfused into culture chambers at the recommended pressure of 2 psi for 3 h at 37°C. Negative controls included bacteria with medium and PI alone. Phase-contrast and fluorescent images of bacteria were captured with a 63 × oil-immersion objective lens every 10-min using a Zeiss Axiovert 200M inverted microscope (Carl Zeiss Inc.) (Khara et al., 2016). Microscopy was performed in the Facility for Imaging by Light Microscopy (FILM) at Imperial College London.

### *In vivo* Evaluation of BM 2 in BALB/c Mice

To evaluate the efficacy of BM 2 *in vivo*, thirteen, 6 to 8-week-old female BALB/c mice infected with H37Rv were used (control group, *n* = 5, plus 3 mice to check bacterial numbers at day 1 post-infection; Treatment BM 2 group *n* = 5). Animals were randomly assigned to 5 per Tecniplast Isocage with *ad libitum* food and water. Mice were infected with 1 × 10^3^ CFU/mouse (35 μL volume) via the intranasal route under isoflurane anesthesia. The following day, 3 mice were culled, the lungs were removed and plated onto agar to determine the inoculum. Animals were monitored daily and weighed on day 7 and on day 15 before treatment began. Treatment of BM 2 was initiated 14 days post-infection as six intra-tracheal doses (5 mg/kg) administered under isoflurane anesthesia on alternate days over 2 weeks. On day 28, the mice were culled by a Schedule 1 method, lungs removed and homogenized, and then plated on to agar for CFU enumeration (Tenland et al., 2018; Tenland et al., 2019). No adverse effects with BM 2 were observed.

## Results

### Peptoid Design Strategy

**FIGURE 1:**
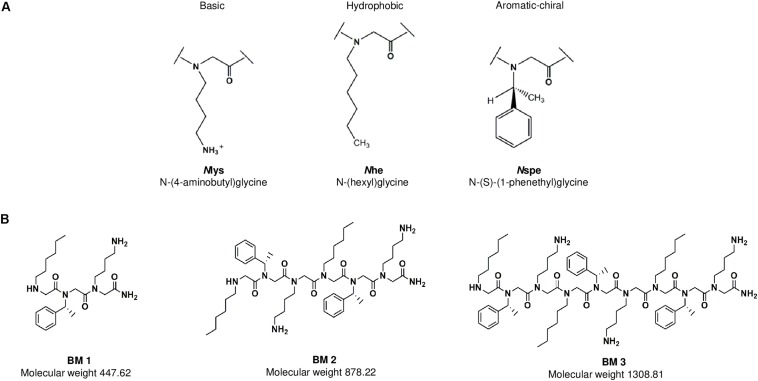
**(A)** The three residues *N*lys, *N*spe, and *N*he comprise one monomeric subunit. These subunits were used to design the three peptoids BM 1, BM 2 and BM 3. **(B)** BM 1 has one monomeric unit (three residues), BM 2 has two repeating monomers (six residues), and BM 3 has three repeating monomers (nine residues).

As peptides are known to be cationic, amphipathic and hydrophobic in nature, peptoids which are non-natural peptidomimetics, would likely need the same characteristics to exhibit antimicrobial properties (Chongsiriwatana et al., 2008; Mojsoska et al., 2015, 2017). Thus, three peptoids (BM 1, BM 2, and BM 3) were designed to study the effect of chain length, charge, and hydrophobicity on antimicrobial activity. Each peptoid contains three residues, *N*lys, *N*he and *N*spe which comprise one monomeric subunit (Figure 1A). BM 1 has a single monomeric unit (three residues), BM 2 has two repeating monomeric units (six residues) and BM 3 has three repeating monomeric units (nine residues) (Figure 1B). The *N*lys residue contributes to increasing the net positive charge of the peptoid, *N*he and *N*spe both contribute to the overall hydrophobicity and *N*spe is aromatic and chiral in nature, while *N*he is less frequently used in peptoid design. These hydrophobic elements were chosen to enhance the anti-TB activity through increasing the interaction with the hydrophobic membrane of *M. tuberculosis*.

### *In vitro* Anti-mycobacterial Activity and Killing Efficacy

The peptoids were assessed for their ability to inhibit the growth *in vitro* of BCG, and drug sensitive and resistant H37Rv and CSU87 *M. tuberculosis* strains respectively, using MIC assays. BM 2 was the most potent against BCG (2–4 mg/L), H37Rv (8 mg/L), and CSU87 (4 mg/L). BM 1 did not display anti-mycobacterial activity against H37Rv or CSU87 at the highest concentration tested (Table 1). MIC was also tested for Gram positive and Gram negative bacteria namely methicillin-sensitive *Staphylococcus aureus* (MSSA), methicillin-resistant *Staphylococcus aureus* (MRSA), *P. aeruginosa, E. coli* and non-tuberculous mycobacteria *Mycobacterium abscessus* and *Mycobacterium avium* but no inhibition was observed (data not shown).

**TABLE 1:** Minimum inhibitory concentrations (MICs) and selectivity indices (SIs) of various peptoids against *M. bovis* BCG, the laboratory strain *M. tuberculosis* H37Rv and the MDR clinical isolate *M. tuberculosis* CSU87.

Peptoid	MIC^a^ (mg/L)			GM^b^	IC_50_^c^	SI^d^	MW^e^	miLogP^f^	R_t_ (min)^g^
	BCG	H37Rv	CSU87						
**BM 1**	16–32	>64	>64	–	>64	n.d	447.6	0.93	8.02
**BM 2**	2–4	8	4	6.3	>64	>10	878.2	2.74	11.51
**BM 3**	4–8	16	16	16	32	2	1308.8	4.54	13.05

*^*a*^MIC results are representative of three independent experiments. ^*b*^The geometric mean (GM) of the MICs against the three mycobacterial strains tested. ^*c*^Cytotoxicity is represented by IC_50_ values, defined as the concentration that inhibits 50% of the metabolic activity of RAW 264.7 macrophage cell line. ^*d*^Selectivity index (SI) is determined as follows: (IC_50_/GM). ^*e*^Molecular weight (g/mol). ^*f*^miLogP (octanol/water partition coefficient) calculated with Molinspiration. ^*g*^Analytical retention times (Rt) estimated on a reverse phase C_18_ Kinetex 100 × 2.1 mm 100 Å column, 60°C run on a 15–65% acetonitrile gradient over 20 min.*

**FIGURE 2:**
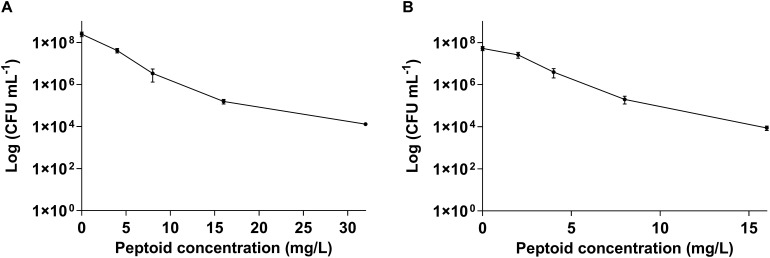
Killing efficiency of peptoid BM 2 against **(A)** drug sensitive *M. tuberculosis* H37Rv and **(B)** multi-drug resistant *M. tuberculosis* CSU87 following treatment for 7 days at concentrations of 4 (0.5 × MIC), 8 (1 × MIC), 16 (2 × MIC), and 32 mg/L (4 × MIC) for H37Rv and 2 (0.5 × MIC), 4 (1 × MIC), 8 (2 × MIC), 16 mg/L (4 × MIC) for CSU87. BM 2 resulted in >99.9% killing of H37Rv at 2 × and 4 × MIC, while >99% and 99.9% killing was achieved against CSU87 at 2 × and 4 × MIC respectively. Data are expressed as mean and standard deviation for three independent experiments.

Further, we tested the bactericidal activity of BM 2 (0.5 ×, 1 ×, 2 ×, and 4 × MIC) against H37Rv and CSU87 for 7 days by CFU enumeration (Figure 2). BM 2 resulted in >99.9% killing of H37Rv at 2 × and 4 × MIC (16 and 32 mg/L), while >99% and 99.9% killing was achieved against CSU87 at 2 × and 4 × MIC (8 and 16 mg/L) respectively, highlighting its bactericidal activity. In comparison rifampicin reduced the bacterial burden by ≥99% at 4 × MIC (0.03 mg/L) after 6 days of treatment as shown by Steenwinkel et al. (2010).

### Cytotoxicity and Cell Selectivity

**FIGURE 3:**
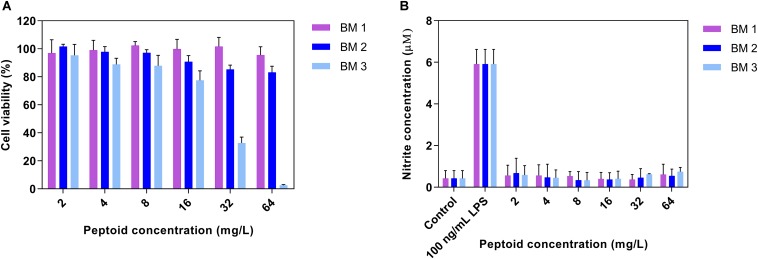
**(A)** Cytotoxicity profiles of peptoids (BM 1, BM 2, and BM 3) and **(B)** the ability of peptoids to promote nitric oxide (NO) production in unstimulated RAW 264.7 mouse macrophage cells following 24 h treatment. Peptoids BM 1 and BM 2 displayed cell viabilities in excess of 80% at 64 mg/L, while the peptoids did not induce NO production as compared to cells stimulated with 0.0001 mg/L lipopolysaccharide (LPS) as the positive control. Control refers to cells treated with media and BM 1-3 alone while LPS treated refers to cells treated with LPS and peptoid BM 1-3. Data are expressed as mean and standard deviation for two independent experiments performed in triplicate.

Peptoids were evaluated for cytotoxicity against RAW 264.7 cells over 24 h in line with our previous work (Khara et al., 2016). BM 1 and BM 2 did not show cytotoxicity at 64 mg/L, while BM 3 showed a 50% reduction in cell viability at 32 mg/L (Figure 3A). The SI of BM 2 and BM 3 was >10 and 2, respectively, but undetermined for BM 1 as it did not have an MIC (Table 1). Hence BM 2 possessed good selectivity for bacterial over mammalian cells.

To establish whether the peptoids stimulate macrophages, production of NO from RAW 264.7 macrophages in the presence of a range of doubling concentrations of peptoids (2 – 64 mg/L) was determined. There was no production of NO from macrophages treated with the peptoids compared to stimulation with the control, LPS (Figure 3B), indicating that these peptoids are unlikely to have any macrophage stimulating activity over the concentration range tested.

### Intracellular Anti-mycobacterial Activity

**FIGURE 4:**
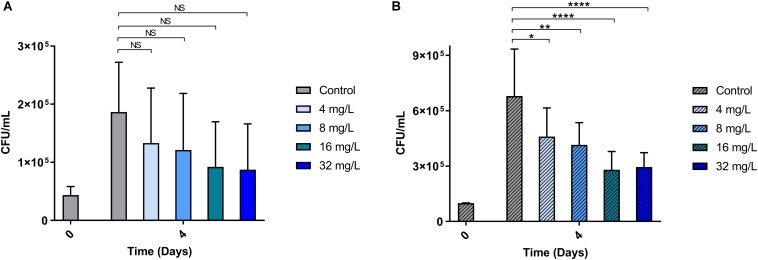
The ability of BM 2 to reduce intracellular bacterial burden (measured as CFU counts) of *M. tuberculosis* H37Rv **(A)** and MDR clinical isolate CSU87 **(B)** after 4 days of treatment. There was a significant reduction in the bacterial burden (between 22 and 56%) for both strains with all concentrations of BM 2 (4, 8, 16, 32 mg/L) compared to the untreated controls as evidenced by the reduction in CFU. The percentage reduction was similar for both H37Rv and CSU87 at each concentration. Data are expressed as mean and standard deviation for two independent experiments. One-way ANOVA followed by Bonferroni’s *post hoc* test was applied for the determination of significant differences where *p < 0.05, **p < 0.01, ****p < 0.0001, not significant (NS).

Intracellular anti-mycobacterial activity was evaluated for the most active peptoid, BM 2 (4, 8, 16, and 32 mg/L), using murine RAW 264.7 macrophages infected with H37Rv and CSU87 for 4 days (Figure 4). Treatment with all concentrations of the peptoid resulted in a similar reduction in mycobacterial burden compared to the untreated control at day 4 (1.8 × 10^5^ CFU/mL for H37Rv and 6.87 × 10^5^CFU/mL for CSU87) for both *M. tuberculosis* strains as determined by CFU counts. BM 2 reduced H37Rv bacterial burden in a dose dependent manner, with a 22% reduction at a concentration as low as 0.5 × MIC (4 mg/L) to 1.36 × 10^5^CFU/mL, and a 51% reduction at a concentration of 4 × MIC (32 mg/L) to 8.75 × 10^4^CFU/mL. A very similar reduction at each concentration was achieved against CSU87 with a 32% reduction at 1 × MIC (4 mg/L) to 4.67 × 10^5^ CFU/mL (*p* < 0.05) and a 56% reduction at a concentration of 8 × MIC (32 mg/L) to 2.96 × 10^5^ CFU/mL (*p* < 0.0001).

### Mechanism of Action Studies: Membrane Permeabilization

**FIGURE 5:**
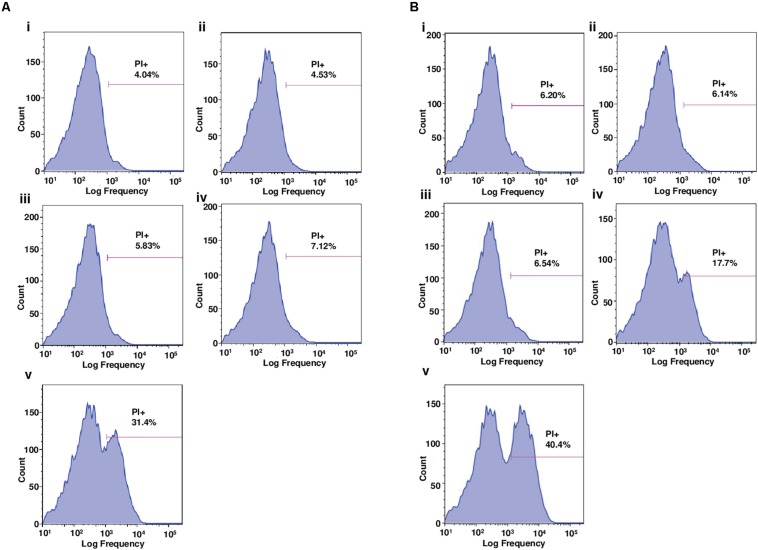
**(A)** Flow cytometric analysis of the proportion of *M. tuberculosis* H37Rv cells positively stained by the membrane-impermeable dye, propidium iodide (PI), following 3 h treatment with **(i)** media, **(ii)** rifampicin, **(iii)** BM 1, **(iv)** BM 2, and **(v)** BM 3. Drug treatments were performed at 4 × MIC concentration (64 mg/L). BM1 treated cells demonstrated negligible uptake of PI similar to the negative controls (media and rifampicin). BM 2 induced a slight uptake of PI and BM 3 induced significant uptake suggestive of membrane permeabilizing mechanisms of action. **(B)** Flow cytometric analysis of the proportion of *M. tuberculosis* CSU87 cells positively stained by PI, following 3 h treatment with **(i)** media, **(ii)** moxifloxacin, **(iii)** BM 1, **(iv)** BM 2, and **(v)** BM 3. Drug treatments were performed at 4 × MIC (32 mg/L) concentration. As expected BM1 treatment resulted in negligible uptake of PI similar to the negative controls (media and moxifloxacin). BM 2 and BM 3 both induced significant uptake of PI suggestive of membrane permeabilizing mechanisms of action. Data are representative from one of three independent runs.

The results from the flow cytometry, showing the proportion of mycobacterial cells fluorescently stained by PI following a 3 h treatment (4 × MIC) with the peptoids or standard anti-mycobacterial drugs (negative controls, rifampicin and moxifloxacin for H37Rv and CSU87, respectively), demonstrate a similar trend for both H37Rv (Figure 5A) and CSU87 (Figure 5B). For H37Rv treated with rifampicin, there was negligible uptake of PI by mycobacteria (4.5%) (Figure 5Aii) similar to medium alone (Figure 5Ai). As expected, the activity of BM 1 was similar to negative controls for H37Rv and CSU87 as this peptoid did not show inhibitory activity in MIC assays, possibly due to its hydrophilic nature (Figures 5Aiii,Biii). BM 2 resulted in a relatively small shift in fluorescence compared to controls (7.12% positive PI cells) (Figure 5Aiv). BM 3 resulted in a significant shift in fluorescence (31.4% positive PI cells), indicative of its membrane permeabilizing activity which could be explained on the basis of its high hydrophobicity which results in this molecule being cytotoxic in nature against both bacterial and mammalian cells (Figure 5Av). CSU87, treated with moxifloxacin (Figure 5Bii) showed PI uptake similar to that of the control (media only) (Figure 5Bi). Both BM 2 and BM 3 caused a shift in fluorescence (17.7 and 40.4% positive PI cells, respectively) and hence it is likely that they both permeabilize the membrane (Figures 5Biv,v).

**FIGURE 6:**
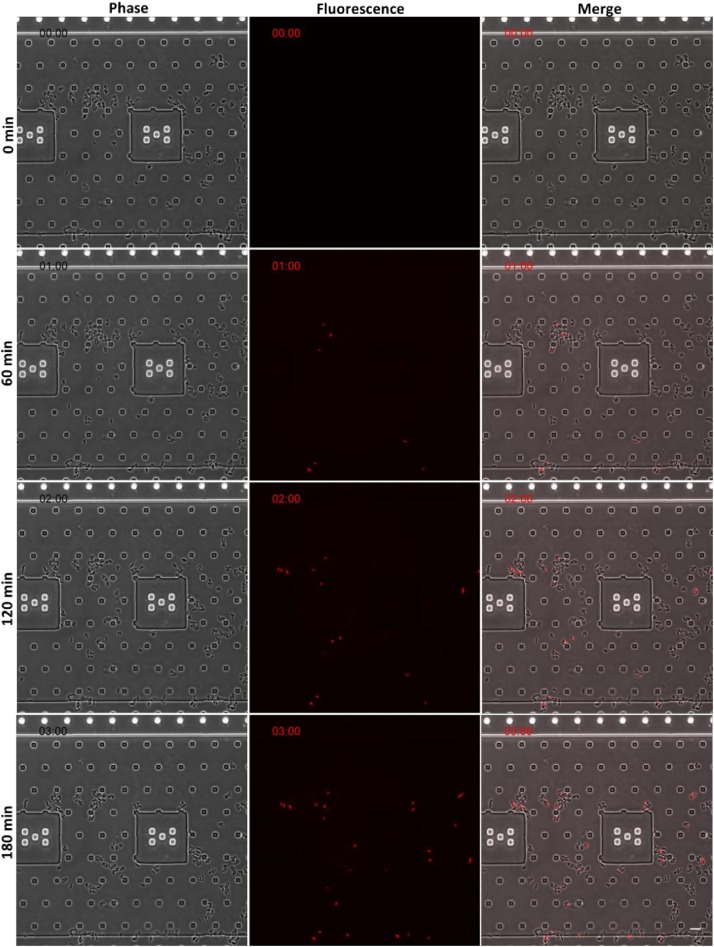
Time-lapse fluorescence microscopy images of BCG (for safety reasons) following 3 h treatment with peptoid BM 2 at 4 × MIC (32 mg/L) in the presence of the membrane-impermeable dye, propidium iodide (PI). Peptoid-mediated membrane disruption promoted uptake of PI into bacterial cells. Images are presented from one representative experiment. Scale bar = 10 μm.

The time-lapse fluorescence microscopy images of BCG (used for safety reasons) (Figure 6), exposed to peptoid BM 2 at 4 × MIC (64 mg/L) for 3 h in the presence of PI, also correlated with the flow cytometry data. Fluorescence staining of bacterial cells demonstrated entry of PI into the cells within 60 min of exposure to BM 2, indicative of membrane disruption induced by this peptoid.

### *In vivo* Efficacy of BM 2

**FIGURE 7:**
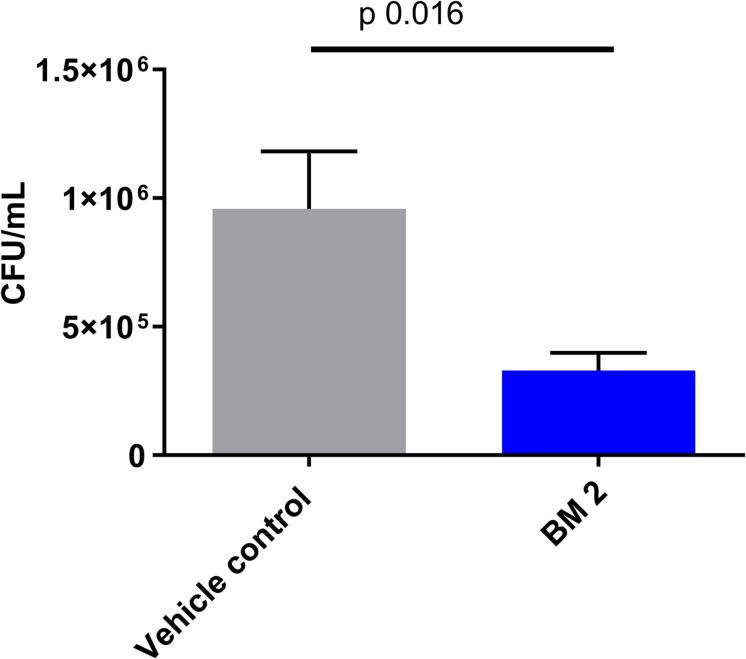
CFU in the lungs of BALB/c mice infected intra-nasally with *M. tuberculosis* H37Rv and treated with six doses of peptoid BM 2 (5 mg/kg per dose) over a 2 weeks period via the intra-tracheal route. The CFU in the lungs of the mice showed a significant reduction in the bacterial load (*p* = 0.016) following treatment with BM 2 compared to the untreated control group. Data is representative of five mice for each group. Mann–Whitney test was applied to determine statistical differences between the two groups.

The *in vivo* efficacy of BM 2 was evaluated in BALB/c mice infected with H37Rv compared to an untreated control group. CFU were estimated in the lung to determine if infection had been established, and whether the peptoid was able to reduce the bacterial burden. At day 1 post-infection, the average infecting dose was 300 CFU/lung (*n* = 3 mice). At 14 days post- peptoid challenge, the CFU counts from the lungs of five mice/group were significantly reduced from 9.5 × 10^5^ in the control group to 3.3 × 10^5^ in the treatment group (∼0.6 log, *p* = 0.016) (Figure 7). As the lung is the primary affected organ in TB, and the peptoid reduced the bacterial load in the organ after only a short treatment period of 2 weeks, we conclude that BM 2 is efficacious *in vivo.*

## Discussion

The mycobacterial envelope is predominantly hydrophobic with a high composition of exceptionally long chain fatty acids known as mycolic acids, and serves as an effective barrier to most broad-spectrum antibiotics. On the other hand, compounds with hydrophobic lipid-like side chains gain easier access by temporarily dissolving in the lipid bilayer of *M. tuberculosis* (Machado et al., 2018). Based on this premise, ultrashort oligo-N-substituted glycines with three monomeric repeating units were synthesized to study the influence of charge (*N*lys), aromaticity and chirality (*N*spe), and hydrophobicity (*N*he), on the potency of the compounds. In comparison to what was reported by Kapoor et al. (2011), where anti-tubercular peptoids were short 4mers with long 13 carbon chains (H-Ntridec-*N*lys-*N*spe-*N*spe-*N*lys-NH_2_), this series of peptoids were ultrashort trimer, dimer and monomer used to investigate chain length and anti-tubercular potency. In addition, preliminary mechanistic evaluations were carried out to delineate their mechanism of action vis-à-vis AMPs.

We employed the whole cell phenotypic screening approach which has been shown to be superior to the target-based approach as evidenced by the discoveries of Bedaquiline and Delamanid (Koul et al., 2011; Laughon and Nacy, 2017). All the peptoids exhibited specificity toward members of the *M. tuberculosis* complex as shown by the lack of activity against Gram positive, negative and non-tuberculous mycobacteria. Among those tested, BM 2 and BM 3 have a net charge of +2 and +3, contributed by the *N*lys residues and believed to be sufficient for initial electrostatic interaction with bacteria. BM 2 and BM 3 also displayed increased hydrophobicity through *N*spe and *N*he groups, providing superior affinity hence anti-mycobacterial activity as compared to the relatively hydrophilic BM 1. Importantly, intracellular bactericidal activity against a drug sensitive and an MDR strain of *M. tuberculosis*, was shown, validating their potential as drugs against intracellular pathogens (Machado et al., 2018). Nonetheless, although BM 3 demonstrated good anti-mycobacterial activity, it displayed reduced selectivity as shown by its increased cytotoxicity to macrophages. This is much to our expectation as reports have already highlighted the balance of hydrophobicity with biocompatibility (Bolt et al., 2017). Both BM 1 and BM 2, on the other hand, demonstrated very little cytotoxic activity (<20%).

AMPs are bactericidal mainly due to their pore forming and membrane disruptive effects resulting in the leakage of cytoplasmic contents out of the bacteria, causing lysis and death (Hancock and Sahl, 2006; Devocelle, 2012). To study the mechanism involved in the bactericidal activity of BM 2 against both H37Rv and CSU87, we investigated the ability of the peptoid to permeabilize or disrupt the mycobacterial membrane in comparison to BM 3, BM 1 and standard anti-mycobacterial drugs. We used flow cytometry, and microfluidics and time lapse microscopy in the presence of PI, a fluorescent DNA intercalating dye that does not penetrate intact bacterial cells; thus it is excluded from live cells and only cells with a damaged membrane are stained (Lin et al., 2009). Bacterial cell fluorescence provides evidence of the membrane-permeabilizing activity of a drug, resulting from the loss of membrane integrity, which allows intracellular diffusion and binding of PI to DNA. The difference in the membrane permeabilizing activity of BM 2 between *M. tuberculosis* strains, may be accounted for by differences in the cell walls, as a consequence of differences in strain origin. Another possibility is that the peptoid may have a non-membrane permeabilizing mode of action. However, we have shown that BM 2 demonstrated bactericidal activity at 2 × MIC against *M. tuberculosis*, with >99% reduction in CFU after 7 days of treatment. Taken together, these findings suggest that the bactericidal activity of BM 2 may partly be mediated by disrupting the structural integrity of the mycobacterial membrane, but other mechanisms may be involved.

BM 2 demonstrated efficacy in the acute model of tuberculosis. Over a period of 2 weeks, treatment with BM 2 reduced the bacterial load in the lungs of the mice. Based on these results future experiments for testing efficacy over a longer duration at different dosages along with pharmacokinetic studies will establish the value of this peptoid as a therapeutic lead candidate.

In summary, this study has shown that peptoids are a promising group of molecules with selective anti-tubercular activity against drug sensitive and MDR *M. tuberculosis*. The selectivity toward bacterial rather than mammalian membranes can be modulated using SAR. The dimer BM 2 showed direct bactericidal activity *in vitro* and efficiently killed drug resistant bacteria at 2 × MIC. It did not activate macrophages, as evidenced by the inability to produce NO, implying that it does not modulate the immune response but rather interacts directly with the bacteria resulting in its bactericidal action on the intracellular bacteria. The mechanism of action is likely to be via disruption of the bacterial membrane as seen by the entry of PI into the mycobacteria. Additionally, BM 2 significantly reduces the bacterial load in the lungs of mice in the acute model of TB. Overall, we have demonstrated that peptoids display anti-mycobacterial activity and thus, in the future, have the potential to be developed into novel therapeutic adjuncts for TB alongside existing drug regimens.

## Data Availability Statement

The raw data supporting the conclusions of this article will be made available by the authors, without undue reservation, to any qualified researcher.

## Ethics Statement

The animal study was reviewed and approved by the UK Home Office (PPL/708653) and all animal procedures were performed in accordance with the Animal Scientific Procedures Act of 1986.

## Author Contributions

JK and BM conducted the *in vitro* experiments. BR and SN conducted the *in vivo* experiments. DM analyzed the data. BM synthesized the peptoids. BM, JK, HJ, SN, PL, BR, and PE designed the experiments. DM, SN, and PE wrote the manuscript. All authors reviewed the manuscript.

## Conflict of Interest

The authors declare that the research was conducted in the absence of any commercial or financial relationships that could be construed as a potential conflict of interest.
