# Supplementary material for: Ultra-Short Antimicrobial Peptoids Show Propensity for Membrane Activity Against Multi-Drug Resistant Mycobacterium tuberculosis
Source: Front Microbiol. 2020 Mar 17;11:417. doi: 10.3389/fmicb.2020.00417 (PMC7089965; doi:10.3389/fmicb.2020.00417)
Supplement: Supplementary file 1 [file Image_1.pdf]

Supplementary Information

A

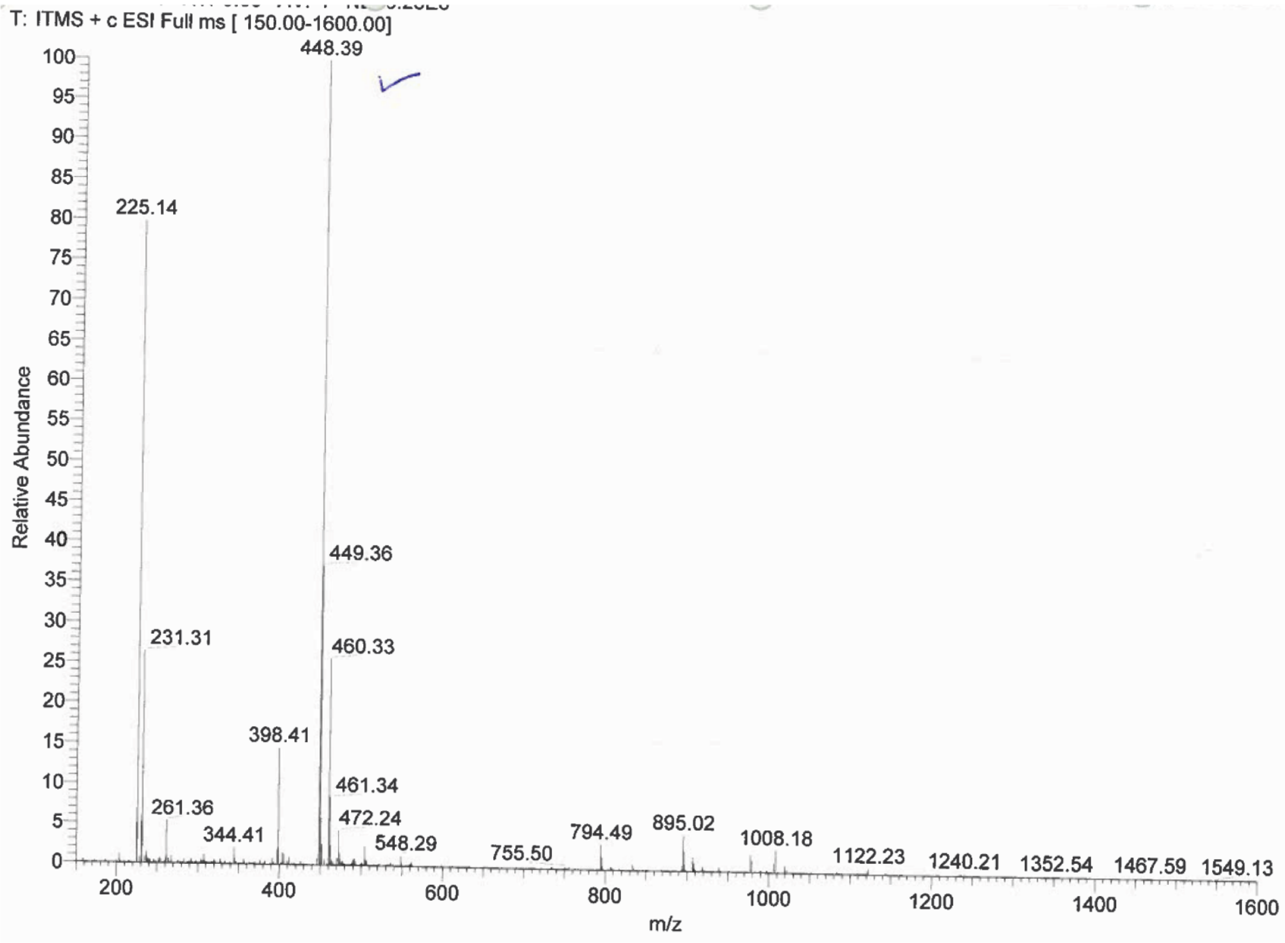

B

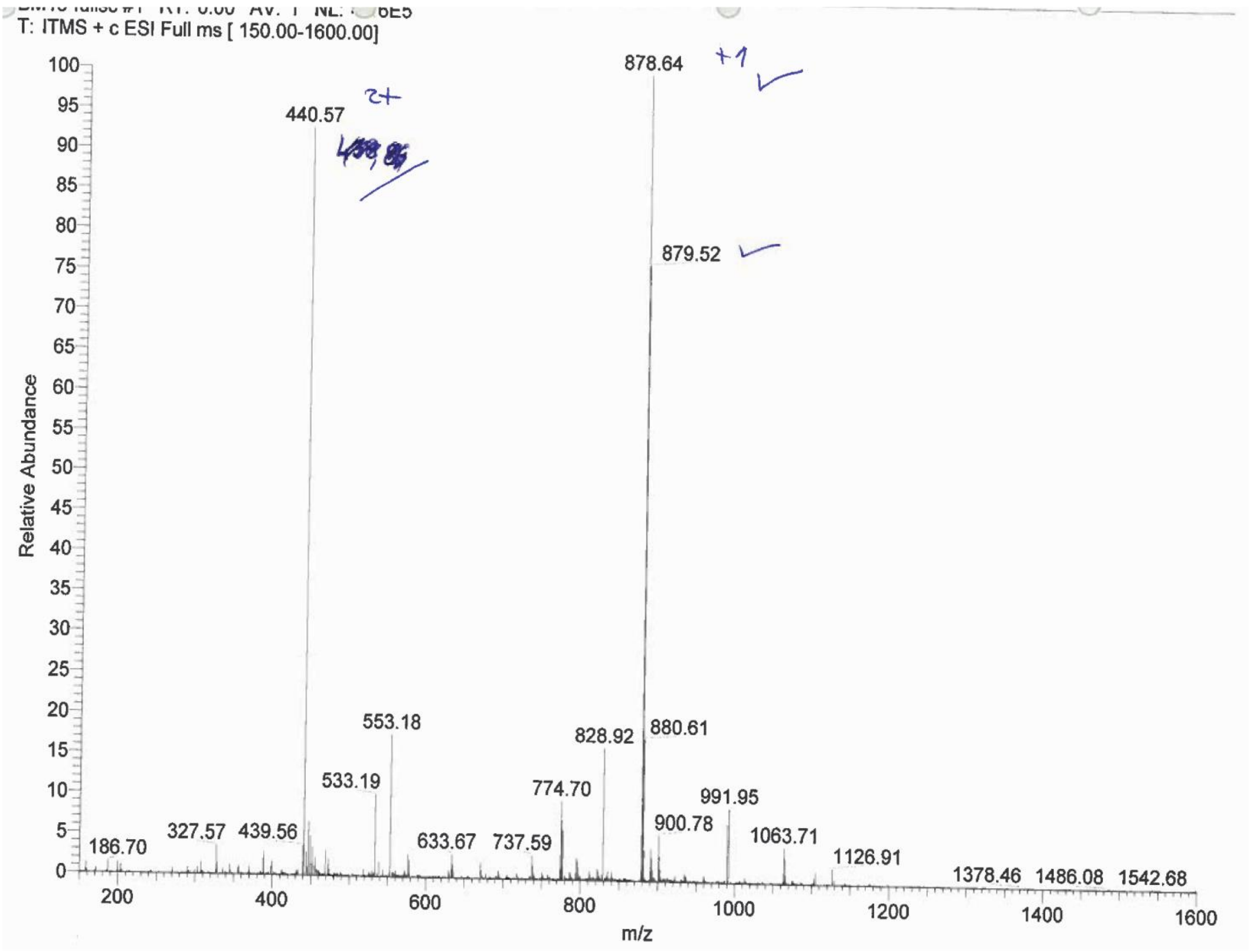
T: ITMS + c ESI Full ms [150.00-1600.00]
Relative Abundance
m/z
878.64
+1
440.57
2+
879.52
553.18
828.92
880.61
533.19
774.70
991.95
900.78
327.57
439.56
633.67
737.59
1063.71
186.70
1126.91
1378.46
1486.08
1542.68

C

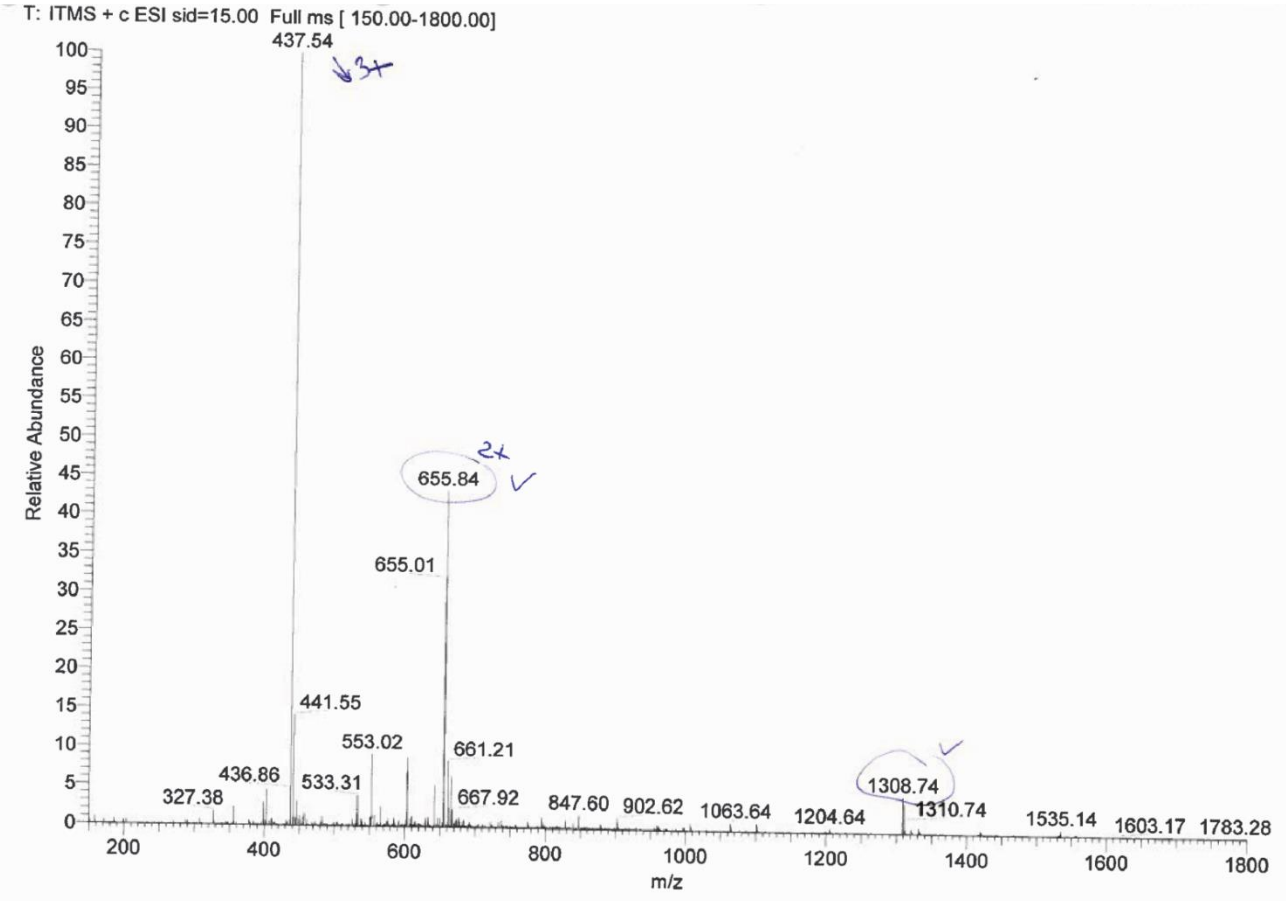

Figure S1. Mass spectrum of peptoids BM 1, 2 and 3. In all chromatograms, the most intensive *m/z* base peak signals are shown corresponding to M+H, M+2H and in some cases M+3H. Peptoid BM 1(A), Peptoid BM 2 (B), peptoid BM 3 (C).
