# Supplementary material for: Ultra-Short Antimicrobial Peptoids Show Propensity for Membrane Activity Against Multi-Drug Resistant Mycobacterium tuberculosis
Source: Front Microbiol. 2020 Mar 17;11:417. doi: 10.3389/fmicb.2020.00417 (PMC7089965; doi:10.3389/fmicb.2020.00417)
Supplement: Supplementary file 2 [file Table_1.pdf]

Supplementary information

Table S1. Sequence and properties of peptoids

Peptoid	Sequence (N-C)	Molecular weight		R_t (min)[a]
		**Observed (*m/z*)**	**Calculated**	
**BM 1**	H-*N*he-*N*spe-*N*lys-NH_2	448.4, 225.1	447.6	8.02
**BM 2**	H-(*N*he-*N*spe-*N*lys)$_2$-NH_2	878.64, 440.6 (M+2H)	878.2	11.51
**BM 3**	H-(*N*he-*N*spe-*N*lys)$_3$-NH_2	1308.7, 655.8 (M+2H), 437.5 (M+3H)	1307.9	13.05

[a] Analytical retention times (Rt) estimated on a reverse phase C_{18} Kinetex 100 x 2.1 mm 100 Å column, 60 °C run on a 15-65 % acetonitrile gradient over 20 min.
